# Impact of Routine Cerebral CT Angiography on Treatment Decisions in Infective Endocarditis

**DOI:** 10.1371/journal.pone.0118616

**Published:** 2015-03-30

**Authors:** Marwa Sayed Meshaal, Hussein Heshmat Kassem, Ahmad Samir, Ayman Zakaria, Yasser Baghdady, Hussein Hassan Rizk

**Affiliations:** 1 Department of Cardiovascular Medicine, Cairo University, Cairo, Egypt; 2 Department of Radiology, Cairo University, Cairo, Egypt; University Medical Center (UMC) Utrecht, NETHERLANDS

## Abstract

**Background:**

Infective endocarditis (IE) is commonly complicated by cerebral embolization and hemorrhage secondary to intracranial mycotic aneurysms (ICMAs). These complications are associated with poor outcome and may require diagnostic and therapeutic plans to be modified. However, routine screening by brain CT and CT angiography (CTA) is not standard practice. We aimed to study the impact of routine cerebral CTA on treatment decisions for patients with IE.

**Methods:**

From July 2007 to December 2012, we prospectively recruited 81 consecutive patients with definite left-sided IE according to modified Duke’s criteria. All patients had routine brain CTA conducted within one week of admission. All patients with ICMA underwent four-vessel conventional angiography. Invasive treatment was performed for ruptured aneurysms, aneurysms ≥5 mm, and persistent aneurysms despite appropriate therapy. Surgical clipping was performed for leaking aneurysms if not amenable to intervention. Results: The mean age was 30.43±8.8 years and 60.5% were males. Staph aureus was the most common organism (32.3%). Among the patients, 37% had underlying rheumatic heart disease, 26% had prosthetic valves, 23.5% developed IE on top of a structurally normal heart and 8.6% had underlying congenital heart disease. Brain CT/CTA revealed that 51 patients had evidence of cerebral embolization, of them 17 were clinically silent. Twenty-six patients (32%) had ICMA, of whom 15 were clinically silent. Among the patients with ICMAs, 11 underwent endovascular treatment and 2 underwent neurovascular surgery. The brain CTA findings prompted different treatment choices in 21 patients (25.6%). The choices were aneurysm treatment before cardiac surgery rather than at follow-up, valve replacement by biological valve instead of mechanical valve, and withholding anticoagulation in patients with prosthetic valve endocarditis for fear of aneurysm rupture.

**Conclusions:**

Routine brain CT/CTA resulted in changes in the treatment plan in a significant proportion of patients with IE, even those without clinically evident neurological disease. Routine brain CT/CTA may be indicated in all hospitalized patients with IE.

## Introduction

Despite the advances in diagnostic and therapeutic techniques, infective endocarditis (IE) remains a challenging disease with high rates of morbidity. Infective endocarditis is fatal if untreated.[1] Clinically manifest neurological complications occur in 20%–40% of IE patients and are mostly due to brain embolization and the formation of intracranial mycotic aneurysms (ICMAs). Additionally, neurological complications in IE are strong predictors of mortality[2,3]; the overall mortality among IE patients with ICMAs is 60% and may reach 80% with ruptured ICMAs[4,5] Autopsy and clinical studies have shown that a significant proportion of cerebral embolizations are clinically silent.[6] Most ICMAs remain silent and rupture suddenly with catastrophic consequences even after apparent bacteriological cure.[7,8] Subclinical infarcts, cerebral hemorrhage and ICMA can complicate the course of treatment and alter treatment decisions, particularly in patients who require cardiac surgery and valve replacement. Despite their high incidence and the dismal prognosis, routine screening for neurological complications is not standard practice in IE. We aimed to assess how routine performance of cerebral angiography would change the treatment decisions in a cohort of IE patients in tertiary care.

## Patients and Methods

The research ethical committee of Kasr Al Ainy approved the research. The local research ethical committee approved the research protocol. We obtained verbal consent from all patients to accept participation in the study. All patients signed a written informed consent before doing CTA, four-vessel cerebral angiography or intervention and before neurosurgery, as this is routine hospital practice. We documented in the patients’ files the elements of the consent form that were presented to the study participants. The consent process was witnessed in all patients by one of the patient’s relative and by a registered nurse. The ethical committee approved the consent forms and consent process.

From July 2007 to December 2012 we prospectively recruited all patients with definite left-sided IE according to modified Duke’s criteria.[9] A neurologist evaluated all patients on admission. The patients were classified as having clinical evidence of brain embolization if they had either new onset of a persistent focal neurological deficit or a transient ischemic attack (TIA) defined as brief episodes of neurological dysfunction resulting from focal cerebral ischemia not associated with permanent cerebral infarction.[10]

Trans-thoracic echocardiography (TTE) was performed using the S5-1 probe of a GE Vivid S5 echocardiography system (GE-Vingmed Ultrasound, Horten, Norway) within 24 hours of admission. Trans-esophageal echocardiography (TEE) was performed using the S7-2 probe of the same machine, if indicated, within 48 hours. All views were standardized according to the guidelines of the American Society of Echocardiography. TTE and TEE data were collected including: valve type; morphology; severity of stenosis or regurgitation; vegetation number, size, shape, mobility and site; ventricular dimensions and systolic function as well as the presence of ring abscesses or intracardiac fistulae.

All patients underwent cerebral angiography within 7 days of admission regardless of the presence of manifest neurological insults. Patients were examined on a 64-MDCT Toshiba Aquilion scanner (Toshiba Medical Systems Europe B.V. Zoetermeer, The Netherlands) using the protocol: collimation, 0.75 mm; rotation time, 0.5 sec; table feed per rotation, 15 mm (pitch, 1.25); and reconstruction parameters 0.75 mm with an interval of 0.4 mm. Scanning was performed from the foramen magnum continuing upward to the centrum ovale. Injection of 90 mL of non-ionic contrast material was started before scanning at a flow rate of 3.5 mL/sec. Bolus tracking was performed with a region of interest placed in the extracranial portion of the internal carotid artery (10-sec monitoring delay). Other parameters were 120 kV, 100 mAs, 512 × 512 pixels, and a field of view of 200 mm. To construct 3D displays, we transferred the images to an independent imaging workstation. Patients with chronic kidney disease were scheduled for cerebral magnetic resonance angiography (MRA) without contrast. All examinations were reviewed and discussed by two neuroradiologists who assessed the presence, number, location and size of aneurysms as well as the associated findings, e.g., vasospasm and occlusions. We diagnosed aneurysms as mycotic in the setting of definite IE and with one of the following criteria:[11]

- The presence of another intra- or extracranial mycotic aneurysm.
- Rupture of the aneurysm.
- Arterial occlusion or stenosis adjacent to the aneurysm.
- Cerebral infarction due to arterial occlusion at the level of the aneurysm.

Patients were referred for cardiac surgery, when indicated, according to the American Heart Association guidelines.[12] We planned to refer patients with ICMA for invasive treatment if they had one of the following:

- ICMA diameter ≥7 mm.
- ICMA diameter ≥5 mm and amenable to endovascular treatment.
- Evidence of leakage around the ICMA.
- Location of the ICMA in the same territory as a symptomatic cerebral infarction.
- Increase in ICMA size on subsequent follow-up studies.

Patients with ICMA <5 mm were scheduled for follow-up angiography after two weeks. If the ICMA regressed or remained stable, then the patient was managed conservatively. When cardiac surgery was performed in patients with ICMAs, it had to be either valve repair or replacement by a biological valve. We also decided to withhold oral anticoagulation in patients with ICMA till ICMA is managed or got thrombosed. We also deferred non-emergency cardiac surgery for 2 weeks in patients with ICMAs even if asymptomatic and in patients with cerebral hemorrhage or large cerebral infarcts even if asymptomatic. We excluded patients who refused to sign the informed consent and patients who could not be referred for angiography within the first week of admission because of logistical reasons.

## Statistical analysis

Numerical variables are described as the mean ±standard deviation. Categorical variables are expressed as percentages. We measured the number of patients in whom we changed the treatment decisions based on the angiography findings as a percentage of the total cohort. Analysis of the data collected was carried out by the Statistical Package for Social Sciences version 17 (SPSS 17.0) program.

## Results

**Fig 1:**
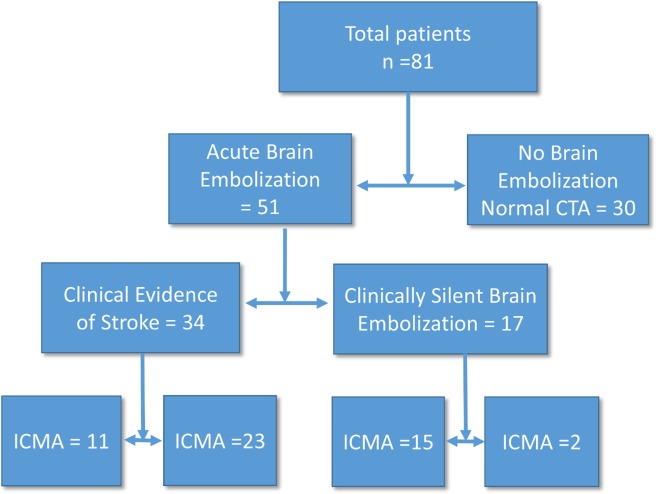
Angiography findings of the studied patients.

From July 2007 to December 2012, 150 patients were referred to the IE working group in Kasr El-Ainy teaching hospitals. Of them 102 consecutive patients had definite left sided IE. Only 81 consecutive patients underwent cerebral CTA/MRA within the first 1 week of admission. CTA was not performed in 21 patients for the following reasons: early death before CTA was performed (n = 7), refusal to participate (n = 12) or other logistical problems (n = 2). Eighty-one patients underwent cerebral angiography within 7 days of admission: 80 had CTA and 1 patient, with chronic kidney disease, had MRA without contrast. The mean age was 30.43±8.8 years and 60.5% were males. The time from symptom onset until referral was relatively long. Staph aureus was the most common organism. The most common underlying valve pathology was rheumatic heart disease (Table 1). Symptomatic neurologic complications occurred in 34 patients. ICMA occurred in 26 (32%) patients; of them 15 (18.5%) had no neurological manifestations. One patient had a silent brain infarction and another patient had a silent cerebral hemorrhage (Table 2). The angiography findings (Fig. 1) changed the treatment plan in 21 patients (25.6%); eleven of whom were neurologically asymptomatic. In 11 patients, more than one change of the treatment plan was made. These changes were:

- We referred 15 patients for invasive treatment of their ICMAs based on the indications previously mentioned; of them 53.3% were asymptomatic. Thirteen patients (15.9%) were referred for endovascular treatment, of them six patients were neurologically asymptomatic. None of them developed procedure-related complications. Two patients were referred for surgical treatment of ICMAs that were not amenable to endovascular treatment; both were neurologically silent. Neither had any procedure-related complications.
- Anticoagulation was stopped in 3 patients with prosthetic valve IE due to the presence of ICMA, 2 of them were silent.
- In 17 patients we had to change the type of cardiac surgery from mechanical valve replacement to repair or biological valve replacement as they had ICMA or asymptomatic hemorrhage. Eight of them had no neurological manifestations.

**Table 1:** Clinical features.

Variable	N (%)	
Time from symptom onset until presentation (days)	52.6±56.4	
Underlying pathology		
Rheumatic		30 (37)
Prosthetic valve		21 (26)
Normal heart		19 (23.5)
Congenital heart disease		7 (8.6)
Degenerative valve disease		4 (5)
Mitral valve		46 (56.8)
Aortic valve		19 (23.5)
Both valves		13 (16)
VSD, PDA, Ao root		3 (3.7)
Healthcare-associated endocarditis*		25 (30.9)
Organisms		
Staph		20 (24.7)
Fungal		8 (10)
Strept		14 (17.3)
Brucella		8 (10)
Culture/serology negative		15 (18.5)
Other		24 (29.6)
Clinically symptomatic neurological complications		34 (42)
Any Major complication**		63 (77.8)
Sepsis requiring ventilation or vasopressor		23 (28.4)
Systemic embolization other than CNS		49 (60.5)
Heart failure		40 (49.4)
Death		15(18.5)

*defined as, 1. Nosocomial infection: infection contracted ≥ 48 hours after hospital admission. 2. Non nosocomial infection: infection appearing ≤ 48 hours of hospital admission within: a) 1 month of receiving IV cannulation, chemotherapy or dialysis; b) 3 months of admission into an acute care facility, c) any time of admission to a nursing home.

**sepsis, major artery embolization, heart failure, death.

**Table 2:** CT/CTA findings.

CT/CTA findings		95% confidence interval
Normal	30 (37)	
Silent infarction	1 (1.2)	26.49% to 47.51%
Manifest infarction	17 (21)	−1.17% to 3.57%
Silent cerebral hemorrhage*	2 (2,5)	12.13% to 29.87%
Manifest cerebral hemorrhage	20 (24.7)	−0.9% to 5.9%
ICMA	26 (32)	15.31% to 34.09%
Silent ICMA	15 (18.5)	21.84% to 42.16%
ICMA ≥5 mm	7 (8.6)	10.04% to 26.96%
ICMA ≥7 mm	19 (23.5)	2.49% to 14.71%

*one patient had a silent cerebral hemorrhage associated with silent ICMA.

Among the 26 patients with ICMAs, 21 (81%) had their management strategy modified on the basis of ICMA presence. This ratio was 73% in those with silent ICMAs (11 out of 15 cases) where the detection of their asymptomatic ICMAs led to treatment plan modifications. In general, treatment plan modifications were in accordance with the AHA/ACCF recommendations for the treatment and management of IE patients and the AHA recommendations for management of unruptured ICMAs.[12–14]

**Fig 2:**
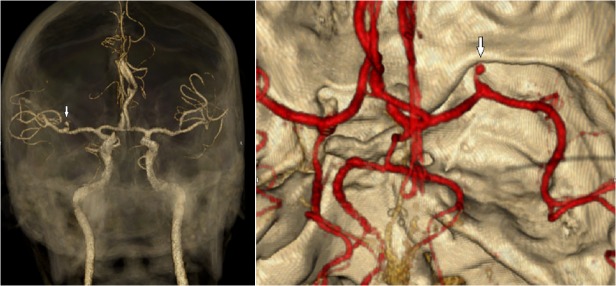
CTA showing a small 3.5 mm ICMA of the right MCA.

Fig. 2 shows the CTA results of a clinically silent ICMA together with its endovascular occlusion.

On follow-up CTA, one patient with multiple ICMAs showed regression in the size of 3 aneurysms and thrombosis of another one; for this patient it was not feasible to perform neurosurgery or endovascular treatment. In another patient a small ICMA 4 mm in diameter showed complete regression. One patient showed thrombosis of a relatively large ICMA of 5.5 mm.

There was no major clinical or laboratory differences between patients in whom treatment plan was modified and others (Table 3).

**Table 3:** Clinical and laboratory characteristics of patients in whom CTA changed the treatment plan.

		CTA effect on decision	
		Did not affect decision	Affected decision
Duration of symptoms before referral (days)		53.8±8	51.4±10
organism identified	unknown	17	4
	staph aureus	11	6
	strept viridans	7	6
	eneterococci	2	1
	Brucella	7	1
	Bartonella	2	0
	Candida albicans	2	0
	aspergillus	4	1
	others	8	4
Vegetations site	mitral	38	10
	mitral & aortic	8	3
	aortic	11	8
heart failure	no severe HF	33	8
	severe HF	27	13
Major complications	non-complicated	10	0
	complicated	50	21
Total		60	21

## Discussion

Our findings suggest that a significant proportion of left-sided IE patients have silent brain embolization that may dismally affect their outcome. Previous studies have been inconsistent regarding the incidence of brain embolization in IE and did not use vascular imaging systematically. Thuny et al.[15] utilized cerebral and thoraco-abdominal CT that revealed silent emboli in a only 8% of patients. Cooper et al performed brain MRI in 40 patients with IE and found acute brain embolization in 80% of patients.[7] Lung et al. performed routine cerebral MRI and detected ischemic lesions in patients 53.3% of their 64 patients.[16] Lung et al also performed routine abdominal and cerebral parenchymal MRI in 58 patients with suspected IE within 7 days following admission. They modified therapeutic plans in 19% of their patients based solely on cerebral MRI, including surgical plan modification in 10% of patients.[17]

Over the past years, we have encountered catastrophic events due to neurological complications in general and ICMAs in particular. Patients without manifest neurological disease can present fatal cerebral hemorrhage weeks after uneventful cardiac surgery and bacteriological cure. We also encountered several patients who bled intracranially waiting for elective cardiac surgery while apparently responding to antimicrobial therapy. Therefore, we decided to perform systematic screening for neurological complications in our referred patients.

To our knowledge this is the first study assessing the routine use of CTA for brain embolization detection in patients with left-sided IE and its impact on treatment plans. ICMAs were the most common clinically silent brain embolization (32.1%). Most of the published data indicate much lower incidence (2–10%).[18,19] We believe that ICMA incidence is underestimated in the literature because no attempts were made to detect silent ICMAs in asymptomatic patients-almost one third of the ICMA cases in this series.

Our findings might have been biased by tertiary center peculiarities that include: long interval from symptom onset to referral, more virulent organisms, high incidence of complications and frequent need for cardiac surgery. Applying a strategy of routine cerebral angiography screening in community settings may reveal different findings. In this study population patients characteristics are different from those of western IE patients [20,21]; however, they are more or less similar to those in publications from the Middle East and some developing countries.[22–25]

Although there are some significant differences from some of the recently published reports on IE [20,21], such as patients being significantly younger and RHD prevails, there are no significant differences from the MRA results reported by Duval et al. In that study routine MRA revealed at least 1 cerebral abnormality in 106 (82%) patients, sixteen of whom had neurologic symptoms. Nineteen (79%) of 114 patients without neurologic symptoms had cerebral abnormalities in MRA. The main difference in these findings is that Duval et al documented a lower incidence of unruptured ICMAs.

Many published studies refer to the comparably high sensitivity and specificity of both CTA and MRA; however, CTA is much less expensive, easier to perform and much more suitable for critically ill patients.[26–30]

CT angiography is, at least, as accurate as magnetic resonance in diagnosis of intracranial aneurysms. In a study by Goto M et al, the accuracy of CT was similar to that of magnetic resonance in depicting branch vessels arising from the aneurysm sac in the middle cerebral artery.[31] In patients with a negative initial catheter angiogram computed tomography angiography had a higher yield than magnetic resonance demonstrating a causative cerebral aneurysm in 9.3% of patients.[32] Magnetic resonance angiography is more susceptible to artifacts and thus re-formatted surface-shaded volume-rendered 3-dimensional images of aneurysms from MRA might be inferior compared to those from CTA.[33]

MRA might be more sensitive for small embolic events but theses won’t have an impact on treatment plan in patients with definite/or possible IE.

In this study, mortality in ICMA patients (19%) was significantly lower than in most of the published data, which show rates approaching 60%.[34,35]. We assume that this might be related to the early detection and treatment of these ICMAs before disastrous rupture occurs. The AHA guidelines on indications for neurovascular intervention provide some support to the practice of treating intracranial aneurysms before rupture if they are amenable to endovascular treatment.[14]

## Conclusion

Routine assessment of aortic/mitral IE patients by cerebral angiography can change the treatment plan in a significant proportion of patients referred to tertiary care. This screening method is safe and may potentially improve the outcome of IE patients. We recommend future studies to compare a strategy of routine angiography versus standard care.
